# Effect of glycaemic control on complications following cardiac surgery: literature review

**DOI:** 10.1186/s13019-018-0700-2

**Published:** 2018-01-17

**Authors:** M. Navaratnarajah, R. Rea, R. Evans, F. Gibson, C. Antoniades, A. Keiralla, M. Demosthenous, G. Kassimis, G. Krasopoulos

**Affiliations:** Oxford Heart Centre, John Radcliffe Hospital, Headley Way, Oxfordshire, OX3 9DU UK

**Keywords:** Diabetes, Cardiac surgery, CABG

## Abstract

**Introduction:**

No uniform consensus in the UK or Europe exists, for glycaemic management of patients with Diabetes or pre-diabetes undergoing cardiac surgery.

**Objective:**

[i] Determine the relationship between glycaemic control and cardiac surgical outcomes; [ii] Compare current vs gold standard management of patients with Diabetes or pre-diabetes undergoing cardiac surgery.

**Methods:**

Searches of MEDLINE, NHS Evidence and Web of Science databases were completed. Articles were limited to those in English, German and French. No date limit was enforced.13,232 articles were identified on initial literature review, and 50 relevant papers included in this review.

**Results:**

No national standards for glycaemic control prior to cardiac surgery were identified. Upto 30% of cardiac surgical patients have undiagnosed Diabetes. Cardiac surgical patients without Diabetes with pre-operative hyperglycaemia have a 1 year mortality double that of patients with normoglyacemia, and equivalent to patients already diagnosed with Diabetes. Pre- and peri-operative hyperglycaemia is associated with worse outcomes. Evidence regarding tight glycaemic control vs moderate glycaemic control is conflicting. Tight control may be more effective in patients without Diabetes with pre−/peri-operative hyperglycaemia, and moderate control appears more effective in patients with pre-existing Diabetes. Patients with well controlled Diabetes may achieve comparable outcomes to patients without Diabetes with similar glycaemic control.

**Conclusions:**

Pre / peri-operative hyperglycaemia is associated with worse outcomes in both patients with, and without Diabetes undergoing CABG. This review supports the pre-operative screening, and optimisation of glycaemic control in patients undergoing cardiac surgery. Optimal glycaemic management remains unclear and clear guidelines are needed.

## Background

Diabetes is a common life-long health condition and a major risk factor for coronary artery disease. Latest estimates show a global prevalence of 382 million in 2013, with a projected rise to 592 million by 2035 [1]. Average annual increases in insulin dependent Diabetes of 3% worldwide and 4% in Europe [2] are reported. In the United Kingdom [UK] there are currently 3.2 million people diagnosed with Diabetes, while another 630,000 remain undiagnosed [1]. Approximately 26 million [8%] people suffer from Diabetes in the United States [US] population, while an estimated additional 7 million are undiagnosed [3]. Due to the slow onset of non-insulin dependent Diabetes, and long pre-detection period, up to one-half of cases may be undiagnosed [1], and an estimated 80 million US citizens are considered to have pre-diabetes, pre-disposing them to an increased risk of developing overt Diabetes.

UK and worldwide data shows that the proportion of people with Diabetes undergoing isolated CABG surgery has increased by 33% in recent years to 25–40% [4]. These patients face increased morbidity and mortality following cardiac surgery and represent a sizeable medico-economic predicament worldwide. Specific UK guidelines and standards exist regarding medical management of Diabetes and risk implications for cardiovascular disease [5]. In the US guidelines for the glycaemic management of patients with Diabetes, or pre-diabetes undergoing cardiac surgery have been available for nearly a decade. Surprisingly however, no uniform consensus in the UK or Europe exists for the glycaemic management of these patients.

This literature review considers the care and outcomes of patients with Diabetes and pre-diabetes undergoing cardiac surgery. The main purpose of this article is to: a] identify current standards of care of patients with Diabetes / pre-diabetes undergoing cardiac surgery and b] address the question; “what is best glycaemic management of patients with Diabetes / pre-diabetes undergoing cardiac surgery?”. Based on the reviewed published literature related to the care and outcomes of patients with Diabetes and pre-diabetes undergoing cardiac surgery, it is clear that there is a lack of evidence against which institutions can benchmark their glycaemic management.

## Methods

### Literature review

In pursuit of clinical evidence regarding management and outcomes of patients with Diabetes or pre-diabetes undergoing cardiac surgery, an extensive search was performed using the MEDLINE, NHS Evidence and Web of Science databases. The search criteria were: [“Diabetes” OR “hyperglycaemia” OR “hypoglycaemia” OR “HbA_1c_” OR “pre-diabetes” OR “glycaemic control” OR “glucose” OR “blood glucose” OR “insulin”] in title/abstract AND [“cardiac surgery” OR “surgery” OR “coronary artery bypass grafting” OR “CABG” OR “cardiovascular”] in title/abstract. Articles were limited to those in English, German and French. No date limit was enforced.

In total 13,232 articles were initially identified. Duplicates and false positives were removed. Following examination of the remaining titles and abstracts only 148 articles were regarded of relevance to the topic of review. Reference lists of these articles were also screened for any further relevant papers. Fifty papers from this search have been included in this review.

## Results – Literature review

### Diabetes and hyperglyacemia

#### Diabetes

It is established for over a decade that patients with Diabetes undergoing isolated CABG surgery are faced with a higher incidence of operation-related morbidity, mortality and post-procedural angina recurrence [6, 7]. Numerous studies show that patients with Diabetes have a significantly greater risk [up to 44%] of readmission following discharge after CABG [6, 8–12]. This finding is also supported by most recent British national data [13]. Despite this, no specific guidance exists in the UK or Europe, as to the optimal level, or method of achieving adequate glycaemic control in patients undergoing cardiac surgery. In the US, guidelines have been available for almost a decade [12].

#### Hyperglycaemia

Distinct from Diabetes, isolated hyperglycaemia is a long established marker of adverse outcome and increased LOS in numerous diverse clinical settings, in both patients with, and without Diabetes. Effects appear to be “dose-dependent”, as longer duration and higher levels of hyperglycaemia are both associated with increased morbidity and mortality [14]. This relationship is also apparent in patients undergoing CABG surgery [15], following acute myocardial infarction [MI] [16], severe trauma, ischaemic stroke, and in critically ill medical [17–19] and peri-operative neurosurgical patients within the ITU environment. Treatment of hyperglycaemia shows clinical outcome benefit [17, 18, 20], however the optimal range and duration of glycaemic control is unclear and remains controversial.

#### Pre-operative hyperglycaemia in surgical patients

At present no specific guidance exists in the UK or Europe, regarding the detection and management of pre-operative hyperglycaemia in patients undergoing cardiac surgery.

The prevalence of hyperglycaemia amongst hospitalised patients is reported as high as 38%. Newly discovered in-hospital hyperglycaemia is associated with a higher mortality rate [16%] compared with hyperglycaemia for patients with known Diabetes [21, 22], increased short-term morbidity, and also short and long-term mortality following non-cardiac surgery [23, 24].

A retrospective analysis of 60,000 patients undergoing elective non-cardiac surgery from the Cleveland Clinic showed that pre-operative hyperglycaemia [random BG ≥12 mmol/l at pre-operative assessment] in patients without an established diagnosis of Diabetes increased 1 year mortality [23]. Diabetic status significantly altered this relationship and for a given level of pre-operative hyperglycaemia; the risk of 1 year mortality was lower in the Diabetes patient group compared with non-diabetes. A similar relationship was demonstrated between pre-admission hyperglycaemia and increased in-hospital mortality in the ITU setting [25], with therapeutic glycaemic control showing benefit in only those without a diagnosis of Diabetes [18]. These findings prompted the authors to suggest that A] pre-operative hyperglycaemia should be given greater consideration in patients without Diabetes than those already diagnosed with Diabetes, and B] the expected benefits of adequate glycaemic control may be determined by the pre-operative diagnosis of Diabetes [23]. This suggestion may be regarded as counter-intuitive, but emphasises the need to “glucose screen” all patients undergoing cardiac surgery, something that is currently not routine practice in the UK.

In another large study, over 20% of ~ 34,000 non-cardiac surgical patients were hyperglycaemic on admission [fasting BG > 6.1 mmol/l] without having a prior pre-operative diagnosis of Diabetes. In over half of these patients, a subsequent provisional diagnosis of Diabetes was made [26]. Hatzakorzian et al., in a much smaller study of non-cardiac surgical patients showed a prevalence of pre-operative hyperglycaemia of greater than 25% [27]. A study of 7310 patients by Lauruschkat et al. [28], showed that the prevalence of undiagnosed Diabetes in patients undergoing CABG to be 29.6%. This was associated with increased rate of adverse outcomes, including those of cardiac resuscitation, re-intubation and prolonged ventilation. Anderson et al., in a study of 1895 patients undergoing CABG showed that patients not known to have Diabetes, but with an elevated pre-operative fasting BG [≥ 5.6 mmol/l] had double their expected 1-year mortality, and this was equivalent to patients known to have Diabetes [29]. Key studies relating to the effects of hyperglycaemia on outcomes are summarised in Table 1.

**Table 1:** Impact of newly discovered hyperglycaemia on the outcome of patients admitted to hospital

	Aim of Study	Results
Umpierrez et al.^22^ *n = 2030* medical patients	To determine the prevalence of in-hospital hyperglycemia and determine the survival of patients with hyperglycemia with and without a history of Diabetes	Newly discovered hyperglycemia was associated with a higher in-hospital mortality rate compared with those patients with a prior history of Diabetes and patients with normoglycemia. Patients with hyperglycaemia had longer length of hospital stay, a higher admission rate to an intensive care unit and were less likely to be discharged home.
Abdelmalak et al.^23^ *n = 61,536* surgical, non-cardiac surgery patients	To study the hypothesis that pre-operative BG levels and the Diabetes diagnosis status of the patients are related to surgical outcomes	One year mortality was significantly related to pre-operative BG. Hyperglycaemic patients with diagnosed Diabetes displayed a significantly lower 1 yr. mortality than hyperglycaemic patients without Diabetes
Noordzij et al.^24^ *n = 108,593* surgical, non-cardiac surgery patients	To determine the relationship between pre-operative BG levels and peri-operative mortality in non-cardiac and non-vascular surgery	Pre-operative hyperglycemia was found to be associated with increased cardiovascular mortality in patients undergoing non-cardiac and non-vascular surgery
Whitcomb et al.^25^*n* = 2713 ITU patients	To assess the association between hyperglycemia and in-hospital mortality in different ITU departments	Higher mortality was seen in hyperglycemic patients without history of Diabetes in the cardiothoracic and neurosurgical units
Anderson et al.^29^*n = 1895* cardiac surgery patients	To determine whether pre-operative fasting BG is associated with an increased mortality after CABG.	Patients not known to have Diabetes but with an elevated pre-operative fasting BG had a 30 day and a 1-year mortality twice that of patients with normal values, and equivalent to patients known to have Diabetes

#### Pre-operative HbA1c

In a prospective study of 3555 CABG patients, an HbA_1c_ ≥ 8.6% [70 mmol/l] was shown to be an independent risk factor for early adverse outcomes and mortality [10]. The same group when conducting a study of 3201 patients demonstrated an HbA_1c_ ≥ 7.0% [53 mmol/l] to be associated with decreased 5 year survival following CABG, compared to patients having a value < 7.0% [53 mmol/l]**.** More importantly, patients with well controlled Diabetes [HbA_1c_ < 7.0%], could achieve comparable outcomes to those patients without a diagnosis of Diabetes [30]. Alserius et al., also demonstrated significantly reduced 3-year survival, and elevated rates of early superficial wound infection to be associated with HbA_1c_ ≥ 6.0% [42 mmol/l] following CABG [31]. However, two studies [32, 33] have failed to show a relationship between HbA_1c_ and LOS, significant early adverse outcomes, or long-term survival following CABG.

Arguably, the predictive value of pre-operative HbA_1c_ in cardiac surgical patients without Diabetes is less well studied. Hudson et al., in a retrospective observational study of 1474 elective patients showed an HbA_1c_ of ≥ 6% [42 mmol/l] in almost a third of patients [31%]. This was associated with elevated intra-operative BG values, a known predictor of adverse outcomes [34], and in isolation, was shown to be an independent predictor of 30-day mortality [35]. Other studies of patients not known to have Diabetes and undergoing percutaneous coronary, vascular or cardiac [4] surgical interventions, also demonstrated a strong association between the pre-procedural elevated HbA_1c_ [30–58%], and risk of early adverse events. These findings suggest that pre-operative HbA_1c_ assessment will be useful as a screening tool in all patients undergoing cardiac surgery, both those with and without Diabetes.

#### Peri and post-operative hyperglycaemia

Intra-operative hyperglycaemia during cardiopulmonary bypass is an independent risk factor for mortality and morbidity in patients with and without Diabetes [34, 36]. Insulin resistance rather than impaired secretion is considered responsible for this [36]. However, it remains unclear whether hyperglycaemia per se, as opposed to increased insulin resistance, drives adverse outcomes. Furnary et al., proposed that improvement in underlying impaired myocardial glycometabolism was one of the predominant mechanisms underlying the favourable effects of insulin therapy, rather than pure achievement of euglycaemia [8, 9] and this has been subsequently supported by other studies [37, 38]. Overall, peri-operative control of hyperglycaemia via continuous insulin infusion was associated with decreased incidence of deep sternal wound infection, shortened hospital LOS, reduced rates of recurrent ischaemia, improved long-term survival and significantly decreased morbidity [8, 11], in a large number of cardiac surgical patients [> 8000]. As such, it is now a globally accepted standard practice of care, although the precise stringency of control i.e. tight vs. moderate, timing and duration of intravenous therapy remain matters of debate [7, 12].

#### Atrial fibrillation in patients with diabetes

The relationship between Diabetes status and post-operative AF requires clearer definition. Most studies do not show any clear association [6], however, some studies show a decreased AF incidence in patients with elevated pre-operative HbA_1c_ [10, 33, 39]. These studies reflect outcomes from a non-UK population, involving pre-dominantly off pump CABG surgery. The potential protective mechanisms of an elevated HbA_1c_ on post-operative AF are unclear. Kinoshita et al. [39], propose that one plausible explanation is that patients with elevated HbA_1c_ require more insulin for adequate glycaemic control, a therapy which is shown to reduce post-operative AF [8, 40]. In support, Lazar et al. have also demonstrated tighter glycaemic control via intravenous insulin to lower incidence of post-operative AF [11].

#### CABG vs non-CABG cardiac surgery

The majority of evidence reviewed in this paper relates to CABG surgery as opposed to non-CABG surgery. Studies including non-CABG cardiac surgery did not clearly delineate outcomes relating to type of surgery, with the majority of patients having undergone CABG. Therefore it is difficult to draw firm conclusions regarding the relationship between deranged glycaemic control, outcomes and precise type of surgery. It is intuitive to think that the effects of deranged glycaemic control on outcomes, would be most prominent following CABG surgery as opposed to non-CABG surgery, due to the well-recognised and established effects on lipid metabolism, endothelial cell function, coronary artery disease, as well as arterial vascular properties / function although, this remains to be proven. Future studies should focus on defining whether deranged glycaemic control has differing effects on outcomes depending on type of surgery.

### Optimal glycaemic care and barriers to standardisation

A critical factor hindering the establishment of clearly defined glycaemic control guidelines is the lack of consensus on what optimal treatment actually is [7, 14]. Brief consensus was reached following 2001, when the Leuven Surgical Trial demonstrated reduced 1-year mortality among critically ill patients when BG levels were tightly controlled between 4.4–6.1 mmol/l as compared to 10.0–11.1 mmol/l [17]. This study instigated an era of tight glycaemic control for all critically ill patients including cardiac surgical patients. The aim of tight control was reinforced by further studies showing beneficial effects of intensive insulin therapy in surgical, medical [18, 20] and cardiac surgical patients [8, 38]. The Portland Diabetic Project provided strong evidence of the adverse effects of hyperglycaemia in patients with Diabetes undergoing cardiac surgery, using an 8.3 mmol/l cut off target value [8, 38].

The concept of tight glycaemic control in critically ill patients was called into question with the publication of the NICE-SUGAR Study [37]. This study of 6104 patients failed to reproduce the findings of the Leuven Surgical Trial, and in fact demonstrated increased 90-day, all-cause mortality after surgery in the tight control group [37]. In support of these findings more recent studies in CABG patients have either failed to demonstrate beneficial effects with tight control [41–44], or shown superior beneficial effects with moderate control [7.0–9.9 mmol/l] [45].

The recent randomised controlled GLUCO-CABG trial of 302 patients showed no difference in outcomes between intensive or conventional moderate glucose control in CABG patients with Diabetes [46]. However, in patients without Diabetes intensive glucose control was associated with lower complication rate. This reinforces the idea from the Portland Diabetic Project and Cleveland Clinic group of the importance of Diabetic status [pre/peri-operative hyperglycaemia in patients with and without Diabetes] [38]. Possibly a lower BG target is needed for patients without Diabetes, whereas a higher target is permissible for those with Diabetes.

The recently published American multicentre study of 4316 cardiac surgical patients by Greco et al., [47] showed that, increasing hyperglycaemia above 180 mg/dl [10 mmol/l] in patients without Diabetes was associated with worsening outcomes. However, this relationship did not hold for patients with non-insulin treated Diabetes. Adding further complexity, this study demonstrated that in insulin treated group allowing BGs above 180 mg/dl [10 mmol/l] was beneficial, with worsening outcomes when “better” control was achieved.

Inducing unnecessary and dangerous hypoglycaemic events with insulin, historically represented another issue driving reluctance to employ stringent BG control protocols. However, these events are now recognised as being rare and avoidable [3, 46], provided BG is frequently monitored.

The lack of consensus amongst the studies we have analysed in this review may be due to the heterogeneity with regards to treatment of hyperglycaemia, glycaemic control protocols, glucose measurement protocols, the glucose metrics employed, their validity and relevance, as well as the individual population demographics. The best metric of glycaemic control remains a matter of debate, and many have been utilised. Average BG over 3 days [3-BG] is considered a good measure [38, 48]. Studies show that metrics incorporating glucose values over longer time periods have greater prognostic relevance in comparison to isolated glucose measurements from just the first 24 or 48-h of an index event e.g. surgical operation or hospitalisation [49]. Metrics of variability/complexity of the circadian glucose pattern are also proposed to be of greater importance than actual BG levels [50].

### Future targeted therapies

The multiple proposed detrimental downstream pathways of hyperglycaemia / insulin resistance, and positive effects of insulin therapy following cardiac surgery are largely unknown and require further detailed definition [3]. They are not the focus of this review, but they are of importance with respect to the development of future targeted therapies. Altered free fatty acid metabolism, endothelial dysfunction, reduced nitric oxide bioavailability and accumulation of reactive oxygen species are implicated [3]. So too is protein kinase C-dependent vasoconstriction, vascular inflammation and platelet aggregation; as well as advanced glycation products [AGE] driven pro-inflammatory cascades [3]. In addition to the metabolic benefits, improved myocardial recovery following myocardial ischemia and direct improvement of contractile function are thought to occur with insulin therapy. Increasing evidence now suggests that reduction in BG variability, rather than absolute levels, to be a major determinant of the beneficial effects of insulin therapy [50]. Other proposed beneficial mechanisms include; membrane stabilization, anti-arrhythmic effects, improved glucose utilization, improved cardiac output via vasodilation and lowering of total peripheral resistance, and improved immune function [3].

### Improving clinical outcomes

The detrimental effects of hyperglycaemia and Diabetes on cardiac surgery outcomes are well recognised. Despite that, clear treatment guidance is still lacking in UK and Europe and this has to be addressed. It is vital for all disciplines associated with the care of cardiac surgical patients, to engage in addressing the discrepancy in quality of outcomes observed in patients with poor glycaemic control. By looking into this discrepancy in outcomes, a decision needs to be made as to whether this discrepancy is A] acceptable, B] modifiable, and if so how, and C] is enough currently being done to minimise, or potentially abolish it. We feel that the current dogma stating that *“patients with Diabetes have worse outcomes than patients without Diabetes following cardiac surgery”* is potentially wrong, as these patients are currently not receiving best therapy, and this dogma must be challenged.

### Proposal’s for quality service improvement

The extensive evidence reviewed in this article provides a sufficient mandate to commence a national / international initiative to standardise and improve the quality of glycaemic control in patients undergoing cardiac surgery in UK and Europe.

In the US, a national initiative to improve post-operative glycaemic control in cardiac patients has already commenced in the form of a **S**urgical **C**are **I**mprovement **P**roject **[SCIP]** [21, 48]. This initiative involves collection and analysis of specific performance measures relating to glycaemic control in all participating cardiac centres, with subsequent public reporting of outcomes and compliance. In addition the Society of Thoracic Surgeons [STS] have published detailed US practice guidelines relating to pre-, intra- and post-operative glycaemic management of patients with and without Diabetes undergoing cardiac surgery; Table 2 [12]. The STS practice guidelines include: A] active control of BGs < 180 mg/dl[10 mmol/l] for all patients during the intra- and post-operative period B] all patients with Diabetes receive an insulin infusion in the operating room and for at least 24 h postoperatively C] pre-operative HbA_1c_ measurement in all patients with Diabetes and those at high risk of post-operative hyperglycaemia_,_ to optimise glycaemic management, and identify patients requiring more aggressive glycaemic control, D] pre-discharge in-patient education of all patients with Diabetes and E] appropriate follow up and communication with primary care physician.

**Table 2:** Summary of the US STS guidelines for glycaemic control during adult cardiac surgery (2008)

A] active control of BGs < 180 mg/dl[10 mmol/l] for all patients during the intra- and post-operative period
B] all patients with Diabetes receive an insulin infusion in the operating room and for at least 24 h postoperatively
C] pre-operative HbA_1c_ measurement in all patients with Diabetes and those at high risk of post-operative hyperglycaemia_,_ to optimise glycaemic management, and identify patients requiring more aggressive glycaemic control
D] pre-discharge in-patient education of all patients with Diabetes and
E] appropriate follow up and communication with primary care physician

It is inevitable that practice and outcomes around the UK and Europe in relation to patients with Diabetes or pre-diabetes varies between individual treatment centres. However, the formation of national guidelines, standardisation of care, centralised reporting and open sharing of glycaemic performance data is critical to improving future standards of care. Formation of this structure along with a national glycaemic SCIP would also serve to incentivise service improvement. An example of such a novel potential European SCIP is shown in Table 3. In addition the novel care pathway utilised in our unit is shown in Table 4.

**Table 3:** Potential Steps for Facilitating Service Improvement in Diabetic / Pre-diabetic Patients Undergoing Cardiac Surgery

Step 1
Publication of detailed and specific guidelines regarding: Pre-operative screening of all patients undergoing elective cardiac surgery and therapeutic intervention for Diabetes / pre-diabetes
Pre-operative target glycaemic criteria permitting elective surgery e.g. HbA_1c_ < 7.5%
Methods, triggers and duration of intra-operative and post-operative glycaemic control
Post-operative / pre-discharge target criteria of glycaemic control on ITU and ward e.g. blood glucose ≤12 mM pre-discharge
Early post-discharge follow up by family doctor / Diabetes specialist team to ensure ongoing good glycaemic control
Step 2
Establishment of a dedicated cardiac diabetic specialist team in every cardiac surgical unit to facilitate: Pre-, peri-, post-operative and discharge glycaemic control and planning
Patient and professional education at all levels and communication with primary and community care services
Step 3
Establishment of specific national diabetic cardiac Surgical Care Improvement Project (SCIP) Europe-wide to include: Introduction of relevant and appropriate performance quality measures e.g.:-
HbA_1c_ measurement in 100% of elective patients undergoing cardiac surgery
Pre-operative point of care fasting blood glucose of ≤8 mM in 95% of operated patients
Pre-operative HbA_1c_ value of < 7.5% in 95% of elective patients going for cardiac surgery
Median post-operative LOS of diabetic patients ≤1.0 day greater than median postoperative LOS for non-diabetic patients
Pre-discharge blood glucose range of 4–12 mM (day before discharge) in 95% of all patients going for cardiac surgery
Post-discharge review by diabetic specialist nurse or family doctor within 1 week in 95% of patients
Incidence of deep sternal wound infection for diabetic patient within the 95% CI of non-diabetic patient A peri-operative glycaemic control multi-disciplinary working group in every cardiac surgical unit, responsible for monitoring and reporting SCIP adherence and compliance.

**Table 4:** Oxford Heart Centre Diabetes Care Pathway

• Routine pre-operative diabetic screening for *all* cardiac surgical elective patients (HbA_1c_). Via *GP or part of Pre Assessment Clinic(PAC)*
• Routine diabetic screening for *all* cardiac surgical *urgent* in-patients (HbA_1c_)
• Point of care diabetic specialist team review of *all* diabetic, *OR* selectively identified “*High Glycaemic Risk”* cardiac surgical patients
• Automatic / mandatory ITU, ward, point of ITU discharge and pre-hospital discharge diabetic specialist team review of all diabetic, *OR* selectively identified “*High Glycaemic Risk”* cardiac surgical patients
• Automatic / mandatory GP or specialist nurse post-discharge follow up arrangement on agreed day e.g. day 4
• Routine pre-operative blood glucose measurement on admission of *all* surgical patients
• Establishment of a glycaemic control working group responsible for regular monitoring, auditing and presenting glycaemic control performance data
• New standardised Intravenous Insulin protocol for all patients undergoing cardiac surgery and guidelines for management of hyper and hypo-glycaemia

## Future studies

Future studies need to A] define the optimal level, duration and timing of glycaemic control of patients undergoing cardiac surgery B] quantify any potential benefit derived from pre-operative glycaemic control optimisation C] define the optimal glucose metrics for glycaemic control assessment and validate their positive predictive value for adverse events and D] mechanistically interrogate and identify potential therapeutic targets that improve outcomes in patients with Diabetes, pre-diabetes and those with peri-operative hyperglycaemia and E] aim to define the precise relationship between and deranged glycaemic control and outcomes following types of cardiac surgery; CABG vs non-CABG.

Establishing a new culture in which widespread detailed measurement, reporting and analysis of glycaemic control in relation to patient outcomes will enhance our understanding, help to identify and direct avenues for research and ultimately improve practice and outcomes in these patients.

## Conclusions

The incidence of diagnosed Diabetes continues to rise, and in addition high levels of undiagnosed Diabetes and pre-diabetes are reported in surgical patients. Poor glycaemic control is associated with adverse outcomes following cardiac surgery, and the evidence suggests that pre-operative hyperglycaemia in patients without Diabetes carries greater clinical significance; than in patients already with diagnosed Diabetes. These results suggest that implementation of routine pre-operative glycaemic screening should be performed in all patients. There is conflicting evidence regarding the precise stringency of glycaemic control that should be employed in these patients i.e. tight vs. moderate control; and this requires clearer definition. Patients with Diabetes with good glycaemic control can achieve similar outcomes to patients without Diabetes undergoing cardiac surgery. As such, the current dogma stating that *“diabetic patients have worse outcomes than non-diabetic patients following cardiac surgery”* is potentially wrong. It is imperative that we generate national and European guidelines and standardise the care for patients with Diabetes/pre-diabetes undergoing cardiac surgery.

The main findings from this review are summarised in Table 5.

**Table 5:** The summary of the main findings of this review

• The proportion of people worldwide with Diabetes undergoing isolated CABG surgery has increased by 33% in recent years to 25–40%
• The incidence of diagnosed Diabetes continues to rise, and high levels of undiagnosed Diabetes and pre-diabetes are reported in surgical patients.
• Pre- and peri-operative hyperglycaemia is associated with worse outcomes following cardiac surgery
• Evidence suggests that pre-operative hyperglycaemia in patients without Diabetes carries greater clinical significance; than in patients already with diagnosed Diabetes.
• Cardiac surgical patients without Diabetes with pre-operative hyperglycaemia have a 1 year mortality double that of patients with normoglyacemia, and equivalent to patients already diagnosed with Diabetes.
• No uniform consensus in the UK or Europe exists, for glycaemic management of patients with Diabetes or pre-diabetes undergoing cardiac surgery.
• Patients with well controlled Diabetes *may* achieve comparable outcomes to patients without Diabetes with similar glycaemic control.
• This review supports the pre-operative screening, and optimisation of glycaemic control in patients undergoing cardiac surgery.
• The optimal glycaemic management of cardiac surgical patients remains unclear and requires definition
• Clear guidelines relating to the glycaemic management of cardiac surgical patients are needed in the UK and Europe
